# Range extensions of Pacific bone-eating worms (Annelida, Siboglinidae, Osedax)

**DOI:** 10.3897/BDJ.11.e102803

**Published:** 2023-06-30

**Authors:** Gabriella H. Berman, Shannon B. Johnson, Charlotte A. Seid, Robert C. Vrijenhoek, Greg W. Rouse

**Affiliations:** 1 Scripps Institution of Oceanography, University of California San Diego, La Jolla, CA, United States of America Scripps Institution of Oceanography, University of California San Diego La Jolla, CA United States of America; 2 Monterey Bay Aquarium Research Institute, Moss Landing, United States of America Monterey Bay Aquarium Research Institute Moss Landing United States of America

**Keywords:** *COI*, phylogeography, deep-sea, invertebrates, whale-falls, polychaetes, range extension

## Abstract

First described in 2004 off California, *Osedax* worms are now known from many of the world's oceans, ranging from 10 to over 4000 m in depth. Currently, little is known about species ranges, since most descriptions are from single localities. In this study, we used new sampling in the north-eastern Pacific and available GenBank data from off Japan and Brazil to report expanded ranges for five species: *Osedaxfrankpressi*, *O.knutei*, *O.packardorum*, *O.roseus* and *O.talkovici*. We also provided additional DNA sequences from previously reported localities for two species: *Osedaxpriapus* and *O.randyi*. To assess the distribution of each species, we used cytochrome c oxidase subunit I (*COI*) sequences to generate haplotype networks and assess connectivity amongst localities where sampling permitted. *Osedaxfrankpressi*, *O.packardorum*, *O.priapus*, *O.roseus* and *O.talkovici* all had one or more dominant *COI* haplotypes shared by individuals at multiple localities, suggesting high connectivity throughout some or all of their ranges. Low Φ_ST_ values amongst populations for *O.packardorum*, *O.roseus* and *O.talkovici* confirmed high levels of gene flow throughout their known ranges. High Φ_ST_ values for *O.frankpressi* between the eastern Pacific and the Brazilian Atlantic showed little gene flow, reflected by the haplotype network, which had distinct Pacific and Atlantic haplotype clusters. This study greatly expands the ranges and provides insights into the phylogeography for these nine species.

## Introduction

**Figure 1.**
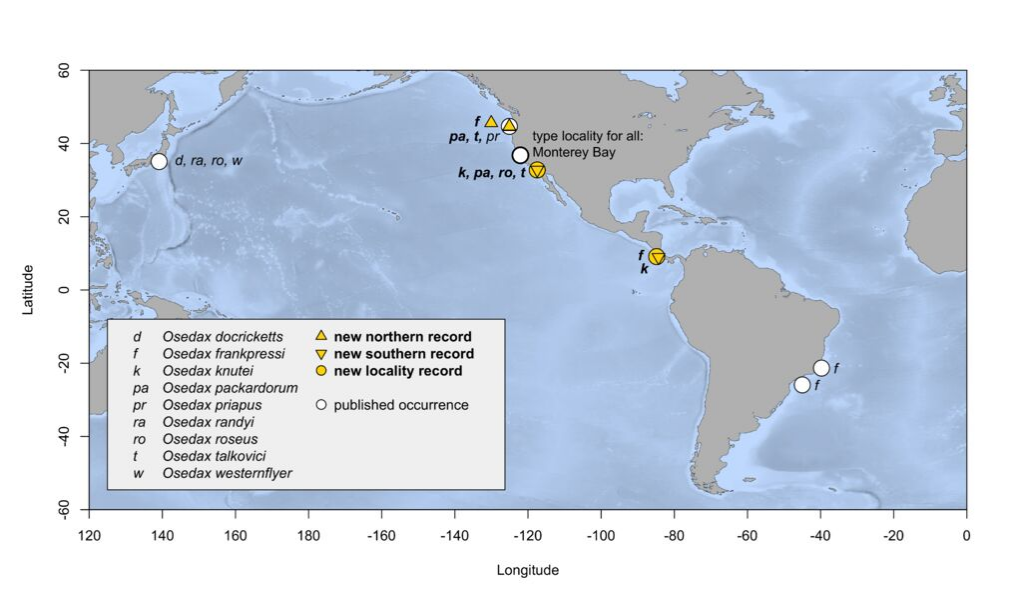
Map of geographic distributions of *Osedax* species analysed in this work. This map was generated using the R package marmap (Pante and Simon-Bouhet 2013).

**Figure 2.**
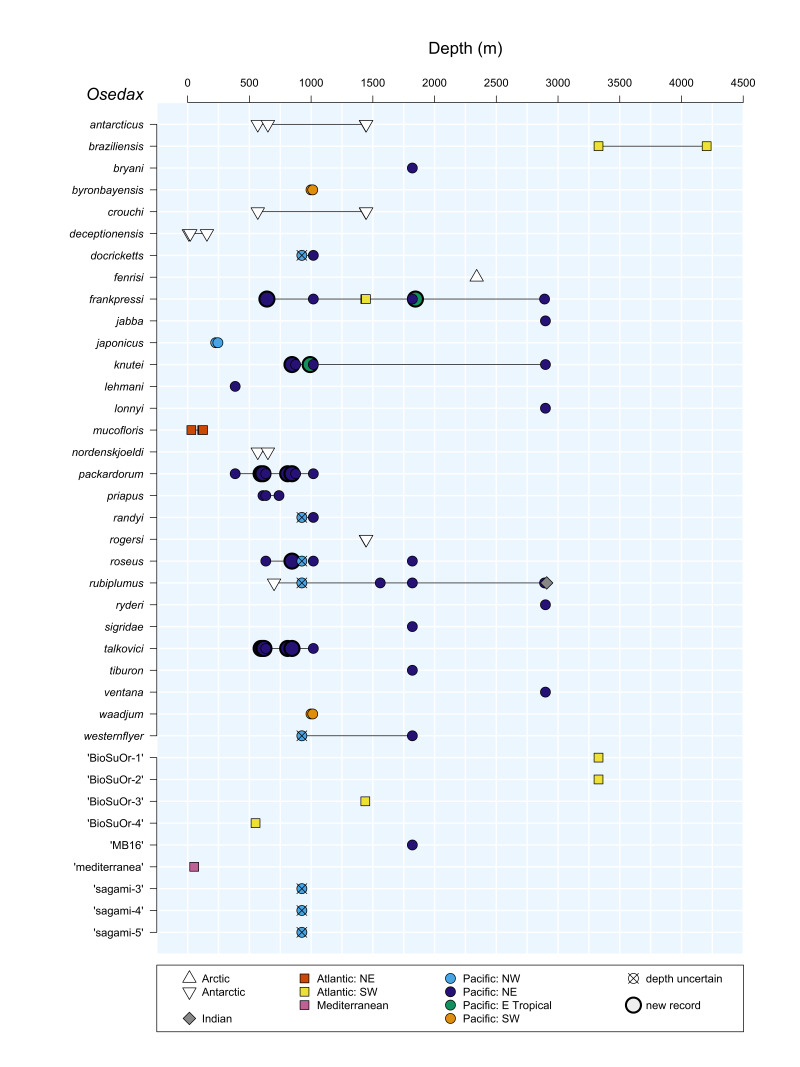
Depth ranges and regions of occurrence for all *Osedax* species reported to date, including undescribed species referenced under informal names. Details and sources are in Suppl. material 1.

*Osedax* (Rouse et al. 2004), part of Siboglinidae, secrete acid to dissolve sunken bone and teeth as a habitat and, aided by symbiotic bacteria, feed on the organic matrix (Rouse et al. 2004, Goffredi et al. 2005, Tresguerres et al. 2013, Rouse and Goffredi 2023). *Osedax* can exploit the remains of diverse vertebrates, from sharks to teleost fishes to mammals, which, together with high fecundity and lecithotrophic larvae may enable them to span extensive ranges (Rouse et al. 2009, Rouse et al. 2018, Zhou et al. 2020, Rouse and Goffredi 2023). To date, 29 *Osedax* species have been formally named, with several others yet to be described (Rouse et al. 2004, Rouse et al. 2018, Fujiwara et al. 2019, McClain et al. 2019, Shimabukuro and Sumida 2019, Eilertsen et al. 2020, Georgieva et al. 2023a); see Suppl. material 1. Most *Osedax* species have only been collected from their type localities (Rouse et al. 2018), though there are a few exceptions (Figs 1, 2); see Suppl. material 1. For example, *Osedaxrubiplumus* (Rouse et al. 2004), originally described from Monterey Bay in central California at 2891 m depth, has subsequently been found in the eastern Pacific, Indian Ocean and Southern Ocean (Zhou et al. 2020). *Osedaxdeceptionensis* was originally described from Deception Island near the Antarctic Peninsula (Glover et al. 2013) and was subsequently recorded from near South Georgia Island in the Subantarctic (Taboada et al. 2015). *Osedaxdocricketts* (Rouse et al. 2018), *O.randyi* (Rouse et al. 2018), *O.roseus* (Rouse et al. 2008) and *O.westernflyer* (Rouse et al. 2018) were all originally described from the eastern Pacific, but are also found in the western Pacific, in Japanese waters (Rouse et al. 2018). *Osedaxpriapus* (Rouse et al. 2015) was originally described from Monterey Bay and Oregon (Rouse et al. 2015). Finally, *O.frankpressi* (Rouse et al. 2004) is known from the eastern Pacific and

the western Atlantic (Rouse et al. 2018, Shimabukuro and Sumida 2019). Much is still unknown about *Osedax* species distributions and the genetic structure across their ranges. In this study, we noted expanded ranges for five *Osedax* species, most of which were previously only known from single localities. We used haplotype networks, based on mitochondrial cytochrome oxidase subunit I (*COI*), to document range extensions and compare phylogeography amongst *Osedax* species.

## Material and methods

We aligned all available mitochondrial cytochrome oxidase subunit I (*COI*) sequence data for *Osedax* from GenBank with new sequences generated from specimens collected from naturally occurring animal falls and experimentally sunken bones off California and Oregon (USA) and off the Pacific coast of Costa Rica (Tables 1, 2). DNA extractions and PCR products were amplified, purified and sequenced following previous protocols (Vrijenhoek et al. 2008, Vrijenhoek et al. 2009).

Alignments for the *COI* data were made in Mesquite (v.3.61) (Maddison and Maddison 2019) using MAFFT with default settings (Katoh and Standley 2013). Uncorrected intraspecific pairwise distances were calculated in PAUP* (v.4.0a168) (Swofford 2002) for each species with untrimmed alignments. Alignments were trimmed to allow for TCS haplotype networks (Clement et al. 2000) to be generated with PopART (Leigh and Bryant 2015). This resulted in alignments of 1005 basepairs (bp) for *O.docricketts*, 462 bp for *O.frankpressi*, 463 bp for *O.knutei*, 793 bp for *O.packardorum*, 891 bp for *O.priapus* (Rouse et al. 2015), 1005 bp for *O.randyi*, 730 bp for *O.roseus*, 807 bp for *O.talkovici* and 983 bp for *O.westernflyer*. The published *O.roseus* sequences EU032471-EU032484 from Monterey were excluded from the *O.roseus* network because there was little overlap with the available Japanese sequences. The published *O.roseus* sequences JF509949 and ON024292 were also excluded from the *O.roseus* network due to sequencing errors at the 5' ends of the sequences. We estimated Φ_ST_ values with Arlequin (v.3.5.2.2) (Excoffier and Lischer 2010) for species with large enough sample sizes; *O.frankpressi*, *O.packardorum*, *O.priapus*, *O.roseus* and *O.talkovici*.

## Data resources

All *COI* sequences in this paper are available on NCBI GenBank, see (Table 2).

## Results

We extended the latitudinal and/or bathymetric ranges for *O.frankpressi*, *O.packardorum*, *O.knutei*, *O.roseus* and *O.talkovici*. *Osedaxknutei*'s range was extended southwards from Monterey Bay (California) to off San Diego (California) and Costa Rica's Pacific coast (Fig. 1). A record of *O.knutei* at 845 m was found in Monterey, expanding the depth range 173 m shallower than previously known (Fig. 2, Suppl. material 1). *Osedaxpackardorum* and *O.talkovici*'s ranges were extended both north and south, from Monterey Bay to Oregon and San Diego (Figs 1, 2). *Osedaxroseus*'s range, previously known from Sagami Bay (Japan) and Monterey Bay, was extended southwards to off San Diego (Fig. 1). *Osedaxfrankpressi*, previously recorded from Monterey Bay and the Brazilian Atlantic, was found off Oregon, establishing a new northern record and also south to Costa Rica's Pacific coast (Fig. 1). The Oregon record of *O.frankpressi* was found at 642 m, expanding the species' minimum known depth by 787 m for a total depth range of 2249 m (642 - 2891 m), representing the widest known range for any *Osedax* species (Fig. 2, Suppl. material 1). An additional sequence was provided from Monterey Bay (type locality) for *O.randyi*. New sequences were provided for *O.priapus* from the two previously reported localities of Oregon and Monterey Bay.

Uncorrected maximum intraspecific pairwise distances ranged from 4.5% for *O.knutei* and 3.9% for *O.frankpressi* to as low as 0.9% for *O.randyi* (Table 3). *Osedaxtalkovici*, *O.roseus* and *O.packardorum* had the largest sample sizes, but not the largest intraspecific pairwise distances (Table 3). Maximum pairwise distances for *O.frankpressi* were 1% amongst samples from the Pacific and 1.7% for the Brazilian Atlantic (Table 3). *Osedaxrandyi* and *O.westernflyer* had the smallest sample sizes and the smallest pairwise distances (Table 3).

**Figure 3.**
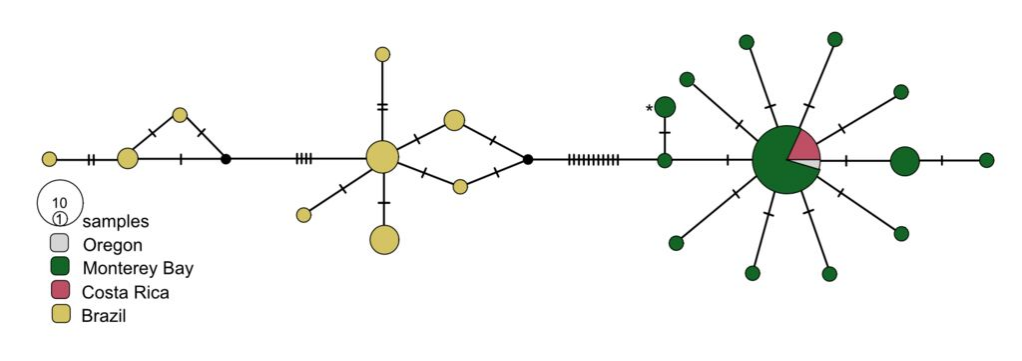
*Osedaxfrankpressi* COI haplotype network coloured by sampling locality. Cross-hatches and black circles represent missing mutations. Holotype haplotype = *. Network made with alignment of 462 bp.

We used TCS haplotype networks of *COI* to visualise the diversity and biogeography of the nine species of *Osedax*. The geographical distribution of *O.frankpressi* was the largest examined, spanning from the Pacific to Atlantic Oceans (Fig. 1). The network for *O.frankpressi* revealed two divergent haplotype clusters, one from Brazil and the other from Oregon, California and Costa Rica (Fig. 3). *Osedaxfrankpressi* differed across its range by nearly 3.9% (uncorrected pairwise distance) and by a minimum of 3% between the Pacific and Brazilian sequences (Fig. 3, Table 3). In the eastern Pacific, one haplotype of *O.frankpressi* was shared from Oregon to Costa Rica and the maximum intraspecific distance was less than 1% (Fig. 3).

**Figure 4.**
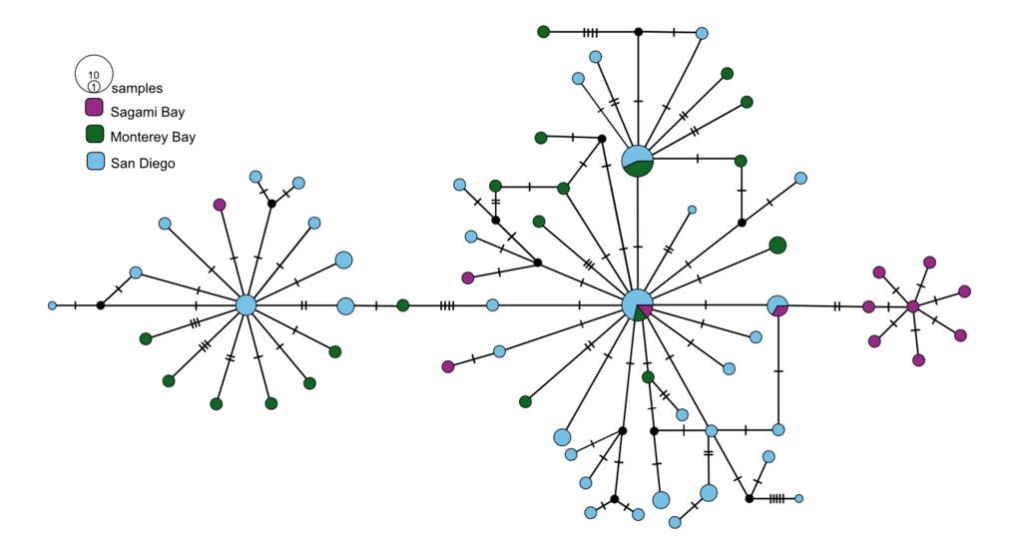
*Osedaxroseus* COI haplotype network coloured by sampling locality. Cross-hatches and black circles represent missing mutations. Network made with alignment of 730 bp.

**Figure 5.**
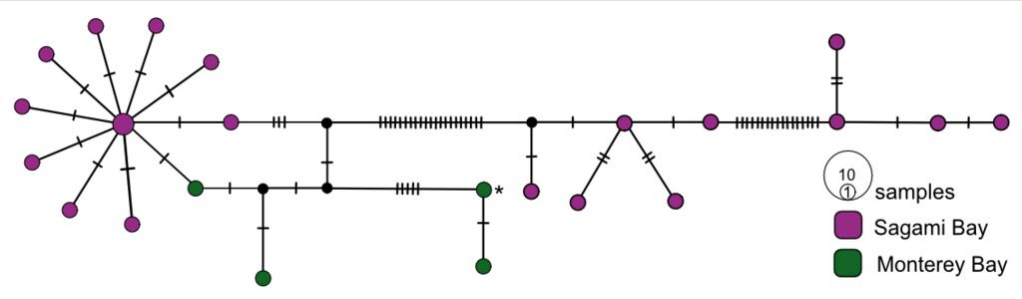
*Osedaxdocricketts* COI haplotype network coloured by sampling locality. Cross-hatches and black circles represent missing mutations. Holotype haplotype = *. Network made with alignment of 1005 bp.

**Figure 6.**
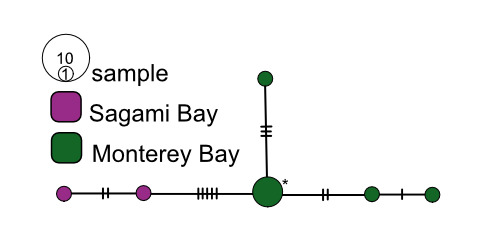
*Osedaxrandyi* COI haplotype network coloured by sampling locality. Cross-hatches and black circles represent missing mutations. Holotype haplotype = *. Network made with alignment of 1005 bp.

**Figure 7.**
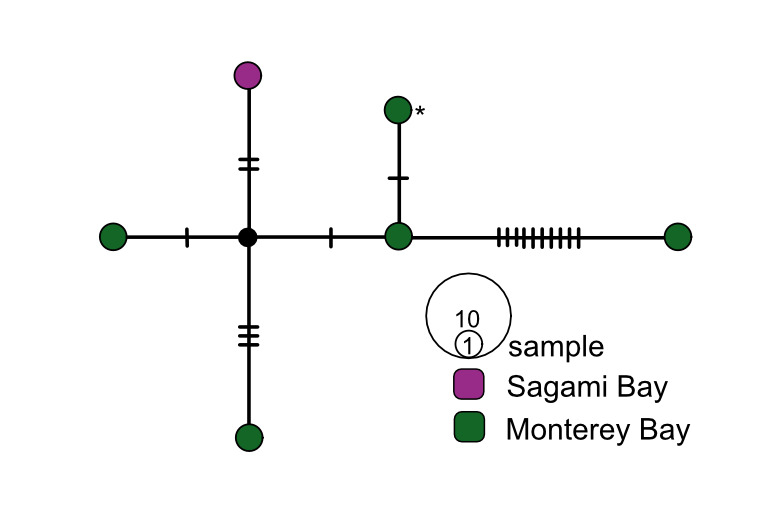
*Osedaxwesternflyer* COI haplotype network coloured by sampling locality. Cross-hatches and black circles represent missing mutations. Holotype haplotype = *. Network made with alignment of 983 bp.

Four species had trans-Pacific distributions. *Osedaxroseus* was found off Japan (Sagami Bay) and California (Fig. 1). Intraspecific diversity was high with three distinct subnetworks, but limited geographic divergence was observed (Fig. 4). Several haplotypes were shared between Japan and California, although a distinct subnetwork was found in Sagami Bay (Fig. 4). Though *O.docricketts*, *O.randyi* and *O.westernflyer* had trans-Pacific distributions (Fig. 1), the limited samples available revealed no shared haplotypes (Figs 5, 6, 7). Haplotype diversity in western Pacific samples of *O.docrickets* was high compared to samples from Monterey and haplotypes were divergent (Fig. 5).

**Figure 8.**
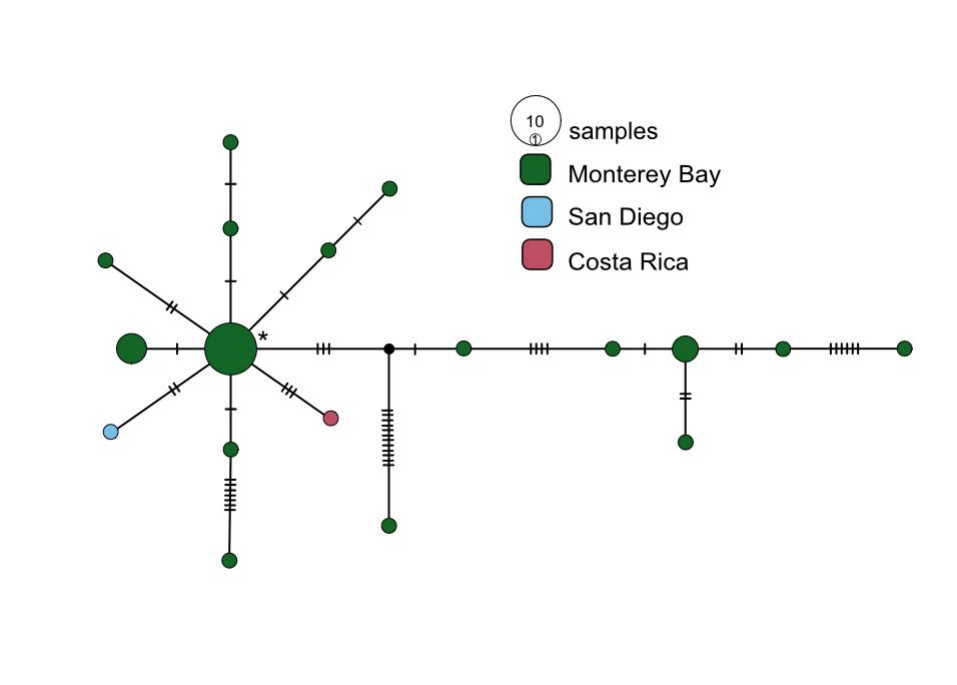
*Osedaxknutei* COI haplotype network coloured by sampling locality. Cross-hatches and black circles represent missing mutations. Holotype haplotype = *. Network made with alignment of 463 bp.

**Figure 9.**
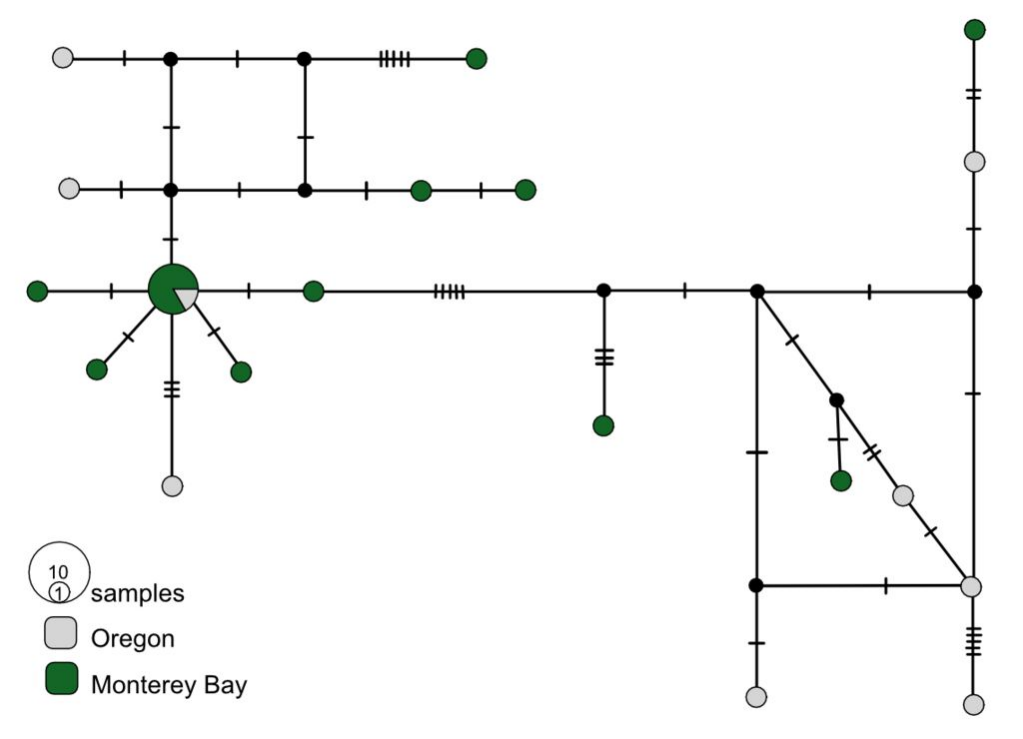
*Osedaxpriapus* COI haplotype network coloured by sampling locality. Cross-hatches and black circles represent missing mutations. Network made with alignment of 891 bp.

**Figure 10.**
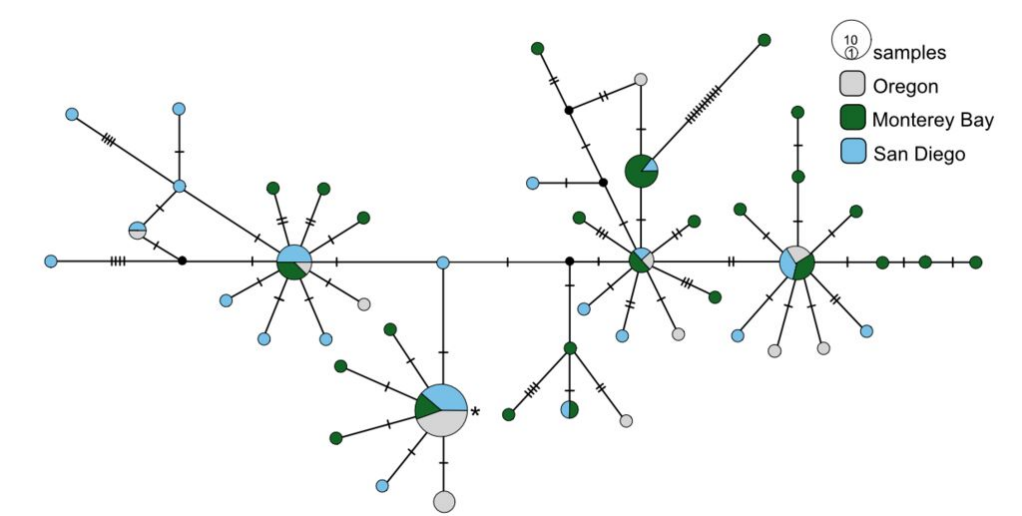
*Osedaxpackardorum* COI haplotype network coloured by sampling locality. Cross-hatches and black circles represent missing mutations. Holotype haplotype = *. Network made with alignment of 793 bp.

**Figure 11.**
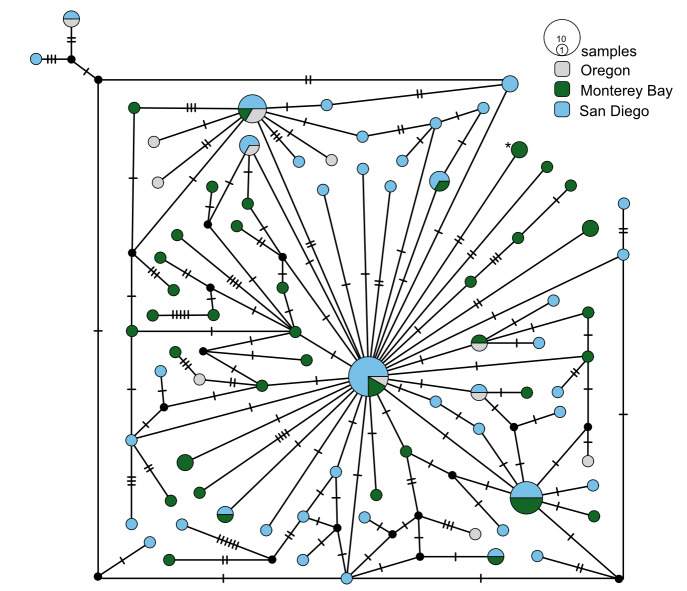
*Osedaxtalkovici* COI haplotype network coloured by sampling locality. Cross-hatches and black circles represent missing mutations. Holotype haplotype = *. Network made with alignment of 807 bp.

Four species have only been found at eastern Pacific locations. *Osedaxknutei* ranged from central California to Costa Rica (Figs 1, 8) and *O.priapus* occured from Oregon to central California (Figs 1, 9). Both species had had similar network topologies with one or two predominant haplotypes and many singleton haplotypes which were somewhat divergent (Figs 8, 9). *Osedaxpackardorum* and *O.talkovici* were distributed from Oregon to San Diego, California (Fig. 1). Both species had many individual haplotypes as well as several haplotypes shared amongst several localities (Figs 10, 11). Each showed some predominant haplotypes shared across most localities (Figs 10, 11). *Osedaxtalkovici* had the largest sample size with 116 sequences and the highest levels of haplotype variability along the eastern Pacific (Fig. 11).

Intraspecific divergence amongst geographical samples was estimated as Φ_ST_ values (Table 4). Most Φ_ST_ values along the eastern Pacific margin were low and not statistically significant (0–0.075), indicating well-mixed populations with high rates of gene flow for all species. However, California and and Brazilian Atlantic samples of *O.frankpressi* were highly divergent (Φ_ST_ = 0.860) and Japan and California samples of *O.roseus* also were significantly divergent (Φ_ST_ = 0.171–0.191).

## Discussion

The data added in this study revealed that many *Osedax* species tend to exhibit higher intraspecific divergence than other siboglinid taxa with comparable ranges (Table 3). For example, the iconic vent vestimentiferan tubeworm *Riftiapachyptila* has a range spanning > 7000 km along the East Pacific Rise, Galapagos Rift and Pacific-Antarctic Ridge from 27°N latitude to 32°S, but *COI* distances are low at ≤ 0.15% (Hurtado et al. 2002, Coykendall et al. 2011). *Tevniajerichonana* has a similar distribution and greater genetic distances (≤ 1.3%) across this range. The western Pacific vestimentiferans, *Lamellibrachiacolumna* and *L.juni*, have comparable intraspecific distances, ≤ 1.24% and ≤ 1.39%, respectively (McCowin et al. 2019). The frenulate *Sclerolinumcontortum* has a bipolar distribution and similar genetic distances, ≤ 1.4% (Georgieva et al. 2015). The vestimentiferans *Escarpialaminata*, *E.southwardae*, *E.spicata* and *E.tritentaculata* show very little *COI* variation across the Gulf of California, the Gulf of Mexico and the Caribbean Sea to the west coast of Africa, with the most common haplotype actually being shared amongst the species (*Cowart et al. 2013, Georgieva et al. 2023b*). The maximum intraspecific distances in named species of *Osedax* ranged from low values of 1.3% in *O.rubiplumus* (*Rouse et al. 2018, Zhou et al. 2020*) and 0.9% in *O.randyi* to highs of 3.5% in *O.docricketts*, 3.9% in *O.frankpressi* and nearly 4.5% for *O.knutei*. Eight out of the nine species considered herein had distances greater than 1.4% (Table 3). The relatively high value obtained for *O.knutei* suggests that the taxon might contain cryptic species and needs further investigation with data from additional genes and samples.

Amongst annelids, the siboglinid clade Vestimentifera appears to be an extreme case of low interspecific distances, as evidenced by the nominal species *Escarpialaminata*, *E.southwardae*, *E.spicata* and *E.tritentaculata*, which actually share a *COI* haplotype, though data from morphology and other genes suggest that they are vaild species on present evidence (Cowart et al. 2013, Georgieva et al. 2023b). Other vestimentiferan interspecific distances can be as low 1.9% between *Lamellibrachiadonwalshi* and *L.judigobini* or 2.5% between *Lamellibrachiabarhami* and *L.anaximandri* (McCowin and Rouse 2018, McCowin et al. 2019, Georgieva et al. 2023b). The smallest interspecific distances observed in *Osedax* to date are 6–7% between *O.randyi* and *O.* 'MB16' and 7.4% between *O.lehmani* and *O.packardorum* (Rouse et al. 2018). Other annelid genera and species with comparable interspecific distances include the dorvilleid *Parougia*, which has minimum interspecific distances of 7% or more (Yen and Rouse 2020), the phyllodocid *Eumidasanguinea* with minimum interspecific distance of 5.5% (Teixeira et al. 2022) and the amphinomid *Eurythoecomplanata* cryptic species complex, with an interspecific distance of 10% in the Atlantic (Barroso et al. 2009). However, there is no clear standard when it comes to species delimitations in annelids. For example, a 5% intraspecific distance was sufficient to split the dorvilleids *Ophryotrocajaponica* and *O.glandulata* (Paxton and Akesson 2010). Nygren (2013) found that minimal interspecific distances of ~ 2 - 23% have been used to delineate cryptic annelid species and distances of ~ 7% are often typical for named congeneric species. This places *Osedax* within the normal minimum interspecific ranges for annelids and makes Vestimentifera somewhat exceptional.

Large geographic ranges in *Osedax* did not always correspond with large intraspecific distances (Table 3). While eastern Pacific samples of *O.frankpressi* differed by up to 3.9% from Brazil Atlantic samples, *O.knutei* had greater intraspecific distances (up to 4.5%) across a range spanning only the eastern Pacific from Monterey to Costa Rica. Similarly, *O.packardorum*, *O.priapus* and *O.talkovici* had relatively high intraspecific distances (2% to 3%) amongst samples from the western margin of the United States. *Osedaxdocricketts* (up to 3.5%) and *O.roseus* (up to 2.4%) both had high intraspecific distances though they have trans-Pacific ranges. *Osedaxrandyi* and *O.westernflyer* also had trans-Pacific ranges, but intraspecific distances were low (≤ 1%). *Osedaxrubiplumus* had the largest known range of any *Osedax*, spanning from Antarctica, across the eastern and western Pacific and the Indian Ocean; yet, its maximum *COI* distance has been recorded at 1.39% (GTR corrected) between California and the Indian Ocean (Zhou et al. 2020).

Despite exhibiting some relatively large geographical distances, *O.packardorum*, *O.priapus*, *O.roseus* and *O.talkovici* exhibited evidence for connectivity across their known ranges. For example, *O.roseus* spans > 8000 km from Sagami Bay and Monterey Bay, as demonstrated by Φ_ST_ values ≤ 0.191. Φ_ST_ for *O.roseus* was 0.00 between Monterey Bay and San Diego, suggesting that the populations might be effectively panmictic. The moniliferan siboglinid *Sclerolinumcontortum* also has a large range, but relatively large sampling has revealed no shared haplotypes between geographical populations (Eilertsen et al. 2018). On the other hand, widely distributed *Osedax* species (*O.packardorum*, *O.priapus*, *O.roseus* and *O.talkovici*) had haplotypes shared across multiple localities further indicating either good dispersal potential across their respective ranges or considerable intermediate habitat (i.e. bones).

Eight *Osedax* species had no haplotypes shared across multiple localities. For *O.randyi* and *O.westernflyer*, the lack of shared haplotypes was likely due to very small sample sizes. Conversely *O.docricketts* and *O.knutei* might encompass cryptic species complexes. For example, nine divergent *O.docricketts* COI sequences occurred in the Sagami Bay, suggesting that cryptic species may occur in Japanese waters, while the real *O.docricketts* may occur in both Sagami Bay and Monterey (Fig. 5). The most divergent *O.docricketts* sequence exhibited 55 nucleotide substitutions from the holotype sequence (asterisk in Fig. 5) (Rouse et al. 2018). In contrast, the *O.talkovici* sample included 116 sequences and had a maximum intraspecific distance of 2.3% (Fig. 11). *Osedaxknutei* had the largest intraspecific distance of any *Osedax* species at 4.5%. The haplotype network for *O.knutei* showed (Fig. 8) that many individuals share a haplotype in Monterey Bay, but there were also divergent haplotypes in Monterey, San Diego and Costa Rica. The large intraspecific distance and the absence of shared haplotypes amongst the three localities suggested that *O.knutei* could be a cryptic species complex, though in sympatry in Monterey Bay.

*Osedaxfrankpressi* and *O.rubiplumus* have the broadest known geographic and depth ranges in this genus (Fig. 2, Suppl. material 1). *Osedaxfrankpressi* also had the largest Φ_ST_ values and one of the greatest intraspecific distances reported in this study (Tables 3, 4). No *COI* haplotypes were shared between the Brazilian Atlantic and eastern Pacific samples; however, one common haplotype was shared amongst Oregon, Monterey Bay and Costa Rica samples. A prior study found ~ 3% divergence between Atlantic (Brazil) and the Pacific (California to Costa Rica) samples, with maximum distances of 0.7% within the Brazil population and 0.3% in the Pacific (Shimabukuro and Sumida 2019). Adding in the new sequences from Oregon, California and Costa Rica samples raised the intraspecific pairwise distances to nearly 3.9%, though the minimum distance between the Brazilian Atlantic and the Pacific remained ~ 3%. The Φ_ST_ value of 0.86 for *O.frankpressi* clearly demonstrated population subdivision between Pacific and Atlantic populations. Although one haplotype was shared amongst samples from Oregon to Costa Rica, a distance of over 6,000 km, further sampling of bones along the east and west coasts of South America might reveal evidence of historical connectivity between Atlantic and Pacific populations, as previously suggested (Shimabukuro and Sumida 2019).

The large ranges for *Osedax* species reported here are not unusual amongst deep sea invertebrates (Georgieva et al. 2015, Eilertsen et al. 2018, Kobayashi and Araya 2018, McCowin and Rouse 2018, McCowin et al. 2019, Shimabukuro and Sumida 2019, Yen and Rouse 2020, Ekimova et al. 2021). For example, the nudibranch molluscs *Dendronotuspatricki* and *D.dalli* and the alvinocaridid shrimp *Alvinocarismuricola* have transpacific distributions comparable with *O.docricketts*, *O.randyi*, *O.roseus* and *O.westernflyer* (Pereira et al. 2020, Ekimova et al. 2021). The siboglinids *Sclerolinumcontortum*, *Lamellibrachiabarhami* and *Escarpiaspicata*, the dorvilleids *Parougiabatia* and *P.billiemiroae*, the maldanid *Nicomachelokii* and the several hesionids belonging to *Sirsoe* or *Vrijenhoekia* have distributions comparable or greater than *O.frankpressi*, *O.knutei*, *O.packardorum*, *O.priapus*, *O.roseus* and *O.talkovici* (Georgieva et al. 2015, Eilertsen et al. 2018, Kobayashi and Araya 2018, McCowin and Rouse 2018, Yen and Rouse 2020, Shimabukuro et al. 2021). For *Osedax*, these large ranges may be conditional on abundant suitable habitats not limited to sunken whale bones (e.g. fish bones etc.), along with high fecundity and lecithotrophic larvae that enhance dispersal capabilities (Rouse et al. 2009, Miyamoto et al. 2013). While it is clear that many *Osedax* species are known to be widely dispersed, the large number of species found in Monterey Bay is interesting (Rouse et al. 2004, Vrijenhoek et al. 2009, Rouse et al. 2015, Rouse et al. 2018). Perhaps other deep-ocean canyons will reveal comparable species diversity as exploration and sampling increase worldwide. *Osedax*'s life history traits make them well suited to wide oceanic dispersal and ecological success. As this study demonstrates, a number of *Osedax* species are as widely distributed as other deep-sea invertebrates that experience little population subdivision across their ranges.

## Supplementary Material

4A4D3E38-53D7-5281-A1AB-BB8DD71A13DB 10.3897/BDJ.11.e102803.suppl1 Supplementary material 1 Supplementary Table 1 Data type occurrences Brief description Geographic and bathymetric occurrence records of all *Osedax* species known to date from peer-reviewed literature and GenBank sequences, including undescribed species referenced under informal names. File: oo_854483.xlsx https://binary.pensoft.net/file/854483 Charlotte Seid and Greg Rouse

## Figures and Tables

**Table 1.** Number of *COI* sequences of *Osedax* used in this study and number of samples from each locality. Range extension = *.

Species	Total	Sagami Bay	Oregon	Monterey Bay	San Diego	Costa Rica	Brazil
*O.docricketts*	24	20	0	4	0	0	0
*O.frankpressi*	54	0	1*	32	0	4*	17
*O.knutei*	34	0	0	32	1*	1*	0
*O.packardorum*	92	0	22*	38	32*	0	0
*O.priapus*	24	0	9	15	0	0	0
*O.randyi*	9	2	0	7	0	0	0
*O.roseus*	85	14	0	19	52*	0	0
*O.talkovici*	116	0	13*	41	62*	0	0
*O.westernflyer*	6	1	0	5	0	0	0

**Table 2.** GenBank accession numbers used for the *Osedax* species in this study. Alternative names listed on GenBank are also listed. New sequences are in **bold**. A total of 258 new sequences were included in this study and released on GenBank.

Species	GenBank number	Other GenBank names
*O.docricketts*	EU267675, EU267676, FJ347625, FJ347626, FM998088-FM998107	Nude-palp C Sagami-6
*O.frankpressi*	AY586486-AY586504, DQ996621, EU223312-EU223316, FJ347605-FJ347607, MH616017-MH616034, **OM994437-OM994445**	-
*O.knutei*	FJ347632, FJ347634, FJ347635, MG262305-MG262307, JF509952-JF509955, **ON041066-ON041090**	Nude-palp E
*O.packardorum*	DQ996639, DQ996641, DQ996642, EU223339-EU223346, EU223349-EU223355, FJ431198-FJ431200, FJ431202-FJ431204, FJ347628, FJ347629, **ON023592-ON023656**	Orange collar Sp. 4 SBJ-2006
*O.priapus*	GQ504740, GQ504741, KP119564-KP119571, **OM988386-OM988399**	Pinnules Sp. 16
*O.randyi*	FM998108, FM998109, FJ347610-FJ347615, **OM734777**	White collar Sagami-7
*O.roseus*	DQ996625-DQ996628, EU032469, EU032470, EU164760-EU164770, EU223317-EU223319, FJ347608, FJ347609, FM998064-FM998077, **ON024260-ON024309**	SBJ-2007a Sp. 2 SBJ-2006 Rosy Roseus (Japan)
*O.talkovici*	FJ431196, FJ431197, FJ431201, FJ431205, FJ347616-FJ347621, JF509950, JF509951, MG262310-MG262313, **ON024160-ON024259**	Yellow patch Pinnules
*O.westernflyer*	FM998110, FJ347630, FJ347631, MG262302-MG262304	Nude-palp D Sagami-8

**Table 3.** Uncorrected maximum intraspecific *COI* pairwise distance matrices for *Osedax* in this study.

Species	Uncorrected pairwise distances
*Osedaxdocricketts*	0.03484
*Osedaxfrankpressi*	0.03927
*Osedaxknutei*	0.04466
*Osedaxpackardorum*	0.02991
*Osedaxpriapus*	0.02021
*Osedaxrandyi*	0.00897
*Osedaxroseus*	0.02392
*Osedaxtalkovici*	0.02283
*Osedaxwesternflyer*	0.01393

**Table 4.** Φ_ST_ values amongst localities of *Osedax* species worldwide. Values in **bold** indicate significant differentiation.

Species	Oregon, Monterey Bay	Oregon, San Diego	Monterey Bay, Sagami Bay	Monterey Bay, San Diego	Monterey Bay, Costa Rica	Monterey Bay, Brazil	Sagami Bay, San Diego
*O.frankpressi*	-	-	-	-	-	**0.860**	-
*O.packardorum*	0.074	0.007	-	0.071	-	-	-
*O.priapus*	0.075	-	-	-	-	-	-
*O.roseus*	-	-	**0.171**	0.00	-	-	**0.191**
*O.talkovici*	0.051	0.024	-	0.039	-	-	-
